# Living with a symptomatic rotator cuff tear ‘bad days, bad nights’: a qualitative study

**DOI:** 10.1186/1471-2474-15-228

**Published:** 2014-07-09

**Authors:** Catherine J Minns Lowe, Jane Moser, Karen Barker

**Affiliations:** 1 Physiotherapy Research Unit, Oxford University Hospitals NHS Trust, Nuffield Orthopaedic Centre, Windmill Road, Headington, Oxford OX3 7HE, UK; 2 Oxford University Hospitals NHS Trust, Nuffield Orthopaedic Centre, Windmill Road, Headington, Oxford OX3 7HE, UK; 3 Nuffield Department of Orthopaedics, Rheumatology and Musculoskeletal Sciences, University of Oxford, Nuffield Orthopaedic Centre, Windmill Road, Headington, Oxford OX3 7HE, UK

**Keywords:** Rotator cuff, Shoulder, Qualitative research, Activities of daily living, Coping strategies

## Abstract

**Background:**

Rotator cuff tears are a common cause of shoulder pain. There is an absence of information about symptomatic rotator cuffs from the patients’ perspective; this limits the information clinicians can share with patients and the information that patients can access via sources such as the internet. This study describes the experiences of people with a symptomatic rotator cuff, their symptoms, the impact upon their daily lives and the coping strategies utilised by study participants.

**Methods:**

An interpretive phenomenological analysis approach was used. 20 participants of the UKUFF trial (The United Kingdom Rotator Cuff Surgery Trial) agreed to participate in in-depth semi-structured interviews about their experiences about living with a symptomatic rotator cuff tear. Interviews were digitally recorded and fully transcribed. Field notes, memos and a reflexive diary were used. Data was coded in accordance with interpretive phenomenological analysis. Peer review, code-recode audits and constant comparison of data, codes and categories occurred throughout.

**Results:**

The majority of patients described intense pain and severely disturbed sleep. Limited movement and reduced muscle strength were described by some participants. The predominantly adverse impact that a symptomatic rotator cuff tear had upon activities of daily living, leisure activities and occupation was described. The emotional and financial impact and impact upon caring roles were detailed. Coping strategies included attempting to carry on as normally as possible, accepting their condition, using their other arm, using analgesics, aids and adaptions.

**Conclusions:**

Clinicians need to appreciate and understand the intensity and shocking nature of pain that may be experienced by participants with known rotator cuff tears and understand the detrimental impact tears can have upon all areas of patient’s lives. Clinicians also need to be aware of the potential emotional impact caused by cuff tears and to ensure that patients needing help for conditions such as depression are speedily identified and provided with support, explanation and appropriate treatment.

## Background

Around 1% of adults aged over 45 years consult their General Practitioner for a new shoulder problem annually; estimations of shoulder pain prevalence range from 4-26%, and rotator cuff problems account for more than two thirds of cases [1]. Shoulder problems are often long term; the majority of people referred to primary care with first episode shoulder pain remain symptomatic one month later and 41% experience persistent symptoms at twelve months [2]. Rotator cuff tears increase with age and may be symptomatic or asymptomatic. 26.2%-38.9% of rotator cuff tears demonstrated during radiological investigations of the shoulder are asymptomatic [3], although tears may become symptomatic over time [4]. Economically, in addition to health care consultation and treatment costs, work related upper limb disorders in the UK are now more prevalent than back pain [5].

The rotator cuff is a critical component of shoulder function and for the successful completion of manual tasks requiring the ability to position the hand precisely in space [6], particularly when the arm is away from the body. There is a lack of concensus regarding the optimal treatment of degenerate cuff tears and limited and inconclusive evidence regarding the relative effectiveness and harms of surgical and conservative treatment approaches [1,7,8]. Non-operative management such as physiotherapy is recommended prior to considering surgery but surgical referral criteria are not straightforward [9,10]. The need for further research was highlighted at a recent consensus meeting on the management of disorders of the rotator cuff which identified 30 unresolved issues/areas for future research to improve management [9].

There is also an absence of information about symptomatic rotator cuff tears from the patients’ perspective which limits the information clinicians can share with patients. The value of qualitative research to improve understanding of patients’ experiences, and of the complex processes involved in treatment outcomes, is well recognized and accepted [11]. One recent Finnish study describes the experience of patients’ diagnosed with supraspinatus tendonitis problems, reporting pain as the predominant attribute of shoulder problems [12] (six focus groups, three pre and three post different types of treatment (n = 21) individuals). More widely, reports include a study briefly exploring patients’ experiences of frozen shoulder and treatment via the Bowen technique [13], a reflection upon the importance of the interpersonal nexus within qualitative research processes with patients undergoing shoulder surgery [14] and a study of patients’ perceptions and priorities regarding frozen shoulder [15]. However, qualitative research regarding rotator cuff tears remains highly limited.

The United Kingdom Rotator Cuff Surgery Trial (UKUFF) was funded by the NIHR Health Technology Assessment Programme to examine the clinical and cost effectiveness of different surgical techniques versus non-surgical treatment for rotator cuff tears. A qualitative study was undertaken to explore UKUFF participants’ experiences of having a known rotator cuff tear and to explore their treatment decision making experiences and outcome. This article aims to describe the experiences of people with a known rotator cuff tear, their symptoms and the impact upon their daily lives and the coping strategies utilised by participants.

## Methods

### Design

A qualitative study using an interpretive phenomenological analysis (IPA) approach [16]. Ethical approval for the study was granted by NRES committee North East - Northern and Yorkshire (ref no: 12/NE/0052) and included obtaining written consent from participants for the publication of their data.

### Participants

Potentially eligible patients were identified by the UKUFF trial team. The inclusion criteria for the UKUFF trial specified patients with full thickness degenerative tears, without trauma, were eligible for inclusion. No patient had an isolated subscapularis tear.

Data collected upon trial entry were used to invite participants with a range of Oxford Shoulder Scores, trial arm allocations, treatments and outcomes. UKUFF participants are English speaking patients aged over 50 with a rotator cuff tear (diagnosed by ultrasound or MRI scanning). Potential participants were posted an invitation by their local UKUFF site principal investigator. Interested patients contacted the study team directly by pre-paid reply slip, telephone or email (their preference) to discuss the study and, if willing, arrange an interview. Written informed consent was obtained pre- interview. Participant characteristics are presented (Table 1).

**Table 1:** Characteristics of study participants

**I/V no**	**Left/right and degree of tear diagnosed by scan**	**Surgery (yes/no, type, size) (if known)**	**Gender**	**Dominant arm**	**Age**	**Lives alone?**
1	R Small/medium	RCR, small	F	Yes	67	No
2	L Small/medium	Yes (post UKUFF)	M	No	63	No
3	R Large/massive	RCR, large and AC jt excision	F	Yes	59	Yes
4	L Small/medium	RCR, massive -	M	No	73	No
5	R Large/massive	RCR, large	M	Yes	68	No
6	L Large/massive	No tear, SAD & AC jt excision	M	Yes	65	No
7	Small/medium	No	M	Yes	56	No
8	R Small/medium	RCR, medium	F	Yes	70	Yes
9	R Large/massive	No	M	Yes	68	No
10	R Large/massive	No	M	Yes	75	No
11	R Small/medium	No	M	Yes	74	No
12	R Large/massive	No	M	Yes	65	No
13	L Small/medium	No	M	No	68	No
14	R Small/medium	No	M	Yes	64	No
15	R Large/massive	No	M	No	68	No
16	L Large/massive	RCR, massive	M	Yes	75	No
17	L small/medium	No	M	No	74	No
18	L Small/medium	RCR, massive	M	Yes	68	No
19	R Small/medium	RCR, medium	F	Yes	54	No
20	R Small/medium USS	Yes (Non UKUFF surgeon)	M	Yes	68	No

Key: I/V = Interview, RCR = rotator cuff repair, SAD - sub acromial decompression, Acromioclavicular Joint excision = AC jt excision. Oxford Shoulder Score = OSS.

#### Sample size

The sample size needed to be sufficiently large to enable relevant data to be obtained, without being so overly large that detailed analysis is subsequently prevented [17]. 20 participants (from 46 people approached) provided a rich insight into the experience of the intervention and recruitment was ceased at this point

### Interviews

A preliminary semi-structured interview guide was developed, following a literature review, by the researchers and an ex-shoulder patient (Table 2). Participants were invited to participate in in-depth semi-structured interviews at a time and venue of their choice. 18/20 interviews were held at participants home and two at conference meeting rooms between September 2012-April 2013. Participants were from across England and Wales, achieving a wide geographical and urban/rural spread. Visits ranged from 50 minutes to two and a half hours in duration, with taped interviews ranging from 28–101 minutes (average 45 minutes). Time was spent after the interviews in everyday conversation, allowing the interviewer (CML) to check the well being of the interviewee [18]; interviews did not cause obvious distress. Interviews were digitally audio recorded and transcribed. Field notes, memos and a reflexive diary were recorded throughout. Participants were posted a summary of their transcript, providing opportunity to check quotations and views and remove anything with which they did not feel comfortable (member checking). No participant withdrew information, several added additional update comments or something they had remembered post-interview.

**Table 2:** Examples of questions asked during interviews

**Question number**	**Question**
1.	Please tell me all about your rotator cuff tear/shoulder? (follow up questions to find out when and and how the tear happened and obtain narrative history from participant).
2.	Please tell me about how your shoulder tear affected/affects you? (follow up questions to explore impact upon activities of daily living, work, leisure, relationships, emotional impact, and to explore symptoms).
3.	Are you right or left handed? (follow up to explore dominance, unilateral or bilateral shoulder problems, “how is your other shoulder?”).
4.	Please tell me anything else you’d like to tell me about your shoulder and how the tear had affected you?

Prompts were used to encourage conversational flow e.g. how did you feel about that? What happened then? Participants were able to introduce any new topics or issues into the interviews at any time and asked for any additional comments.

### Data analysis

Audio recordings were listened to and transcripts read until they become familiar. Data was coded in accordance with IPA [16]. CML broke down interview data into discrete units and wrote these in the right hand margins of transcripts, making concerted efforts to remain close to the data and continually explore meaning. Units found to be conceptually similar were grouped together under more abstract categories and these written in the left hand transcript margins. NVIVO 9 software was used to assist data management. The process of constantly comparing data, codes and categories occurred throughout all analyses. The first three interviews were considered a pilot phase and the analyses discussed by all authors. The sampling approach was discussed at this point, and again after nine interviews. No changes in approach were considered necessary since a wide range of Oxford Shoulder scores, degree of tear and outcomes were being provided by participants. Further strategies to promote rigour, including peer review, code-recode audits, constant comparison of data, codes and categories occurred throughout. KB assisted in the peer review of emerging codes and categories; including independently coding a sample of the interview data (n = 8 full transcripts, including the pilot 3). Literature searches, to promote rich interpretation of the data, were incorporated in the analyses and writing up.

## Results

**Figure 1:**
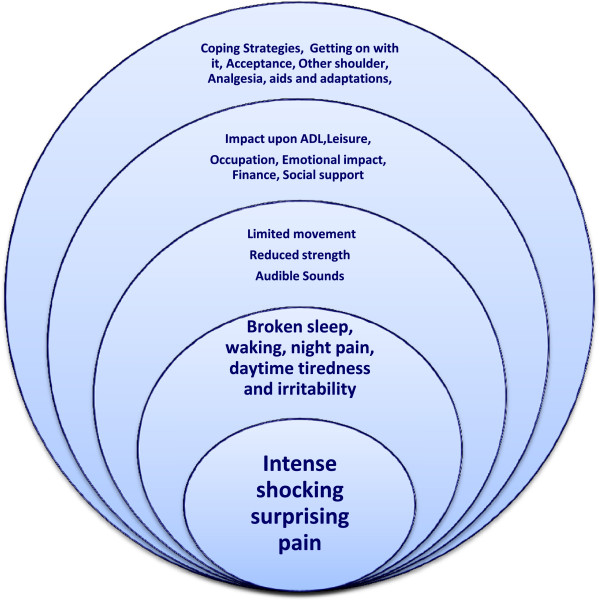
**Diagrammatic summary of living with a rotator cuff tear.** This diagram shows how, like ripples spreading out from a stone thrown into a pool, pain from a symptomatic rotator cuff tear can impact upon, and change, all areas of a participant’s life.

There are three main sections: the identification and description of the symptoms caused by known rotator cuff tears, the impact that these tears had upon the lives of the participants followed by coping strategies that participants used to help them live with their rotator cuff tears. Figure 1 summarises the findings.

### Symptoms

#### Pain

The interplay between peripheral pain detection and central pain processing mechanism systems produce complex perceptions of shoulder pain; sometimes leading to an apparent mismatch between demonstrated pathology and pain perception [19]. Months (in some cases, years) of severe pain was the predominantly described symptom expressed by all participants. The sheer intensity of pain was described by the majority of participants, “excruciating” (I/V 13) “*so* much pain” (I/V 6), “Arghhh!”(I/V 8), so bad “you can’t believe” (I/V 3) and its severity shocked participants:

“I have had (a) back operation, I have fallen out of trees but this pain was far worse than any other pain I have ever had in my life (I/V 14)

The shocking nature of shoulder pain generally is beginning to be recognised [12,15]. The pain “literally stopped you dead in your tracks” (I/V10), “it stopped me in my tracks and made me feel sick (I/V6) “Participants described two main types, an underlying “constant but dull” (I/V 2) “constant nagging pain” (I/V 6) that “just wouldn’t go away (I/V 19) and a seriously intense pain upon/after certain movements:

“when I put my wallet in my back pocket……it was so painful I couldn’t even put my hand there” (I/V12)

“if I do too much with it I think ‘Oh ****’, it’s afterwards it’s burning” (I/V7). Participants explained how the pain “was frightening” (I/V12) and lived with “the fear of that intense pain hitting you again” (I/V 12).

#### Sleep

Most participants repeatedly recounted how sleep was severely detrimentally affected by rotator cuff tears: “I couldn’t sleep on the night” (I/V9). For some, night pain and lack of sleep was “what prompted me to go to the doctors in the first place” (I/V 17). Participants’ expressed problems in getting, and then staying, asleep:

“trying to find a comfortable position to sleep, terrible…. then turn over suddenly and it was painful (I/V11)

“When I was in bed sometimes, my arm would drop out and the pain was really really bad, I would scream out” (I/V15)

Sleep was often broken “if I…. turn the wrong way in bed at night it’ll wake me up” (I/V 15) which “*really* was horrible” (I/V 6). Experimenting with different sleeping positions helped some, “if I lay in a certain position it was more comfortable…..more relaxing” (I/V17) and “I used to have to sleep with a pillow just to keep my arm up” (I/V 19). Lack of sleep affected people’s daytimes too:

“I was really tired. Yeah the lack of sleep…..there’s always pressure at work…..you start getting a little bit edgy, short tempered at times (I/V 5).

Only one participant “never had any trouble sleeping” (I/V 16). Others expressed the need of “getting a good night’s sleep has always been important to me” (I/V 17):

“I’m a person who really needs 8 hours sleep and it was affecting me because if I don’t get my sleep I am irritable and I’m not at my best, let’s put it that way” (I/V 17)

One participant pointed out that his lack of sleep had the knock on effect of:

“keeping my wife awake, I actually slept many times in the spare bedroom because she had to work? I’d go in the spare bedroom and sit there reading a book” (I/V6).

The ability to sleep comfortably is lessened with symptomatic rotator cuff tear [20]. The impact on sleep seems greater than reported by Nyman et al [12] where sleep was disturbed “to varying degrees” but similar to reports of frozen shoulder [15,13].

#### Limited movement

Some participants recounted “pretty hopeless” (I/V 15) and severe restrictions in ability and mobility due to reduced shoulder movement. For some, the lack of movement was influenced by pain:

“I was struggling to use it…..I couldn’t put it (arm) above my head to do anything (*Interviewer: Because of pain or because you didn’t have the movement?*) Um, it was probably both actually…yes” (I/V20).

Others recount movements feeling restricted or tight “it’s limited now (hand behind back) I can feel it” (I/V 14) and “I can feel a bit of pull there” (I/V 17).

Some participants found that movement improved over time:

“after a few months it seemed to get better, I thought, well it doesn’t get better…..some of the muscles just compensate for the damage?” (I/V 7)

Rarely, movement was unaffected. One participant was “what we used to call double jointed” (I/V 8); she was flexible and maintained range of motion but experienced awful pain upon and after movement.

#### Lack of muscle strength

Muscle atrophy is both a known consequence of rotator cuff tear and prognostic factor of outcome following cuff repair [20]. People with non-painful cuff tears demonstrate muscle weakness [21]. The impact that a tear can have upon muscle strength, in terms of muscle weakness rather than pain inhibition, was mentioned by some, but not the majority, of participants; “it’s mainly the weakness” (I/V 18), “I can’t lift any weight” (I/V 9). This was especially true for overhead activities:

“I would have to use the good arm to lift it, I’ve no strength at all in that arm, above about *that* high” (I/V 11)

“I do all my painting….to do a ceiling now I find difficult, because you are working over your head……I have to stop every five minutes to rest” (I/V16).

#### Sound effects

Several participants mentioned the audible sound effects associated with their rotator cuff tear “just listen!” (I/V15), “sometimes I get a crunch, like if I go like *that* it clunks” (I/V 14), “it makes a creaking noise” (I/V18). Not all noises were painful, but were a reminder of their shoulder problem, although a few participants equated worsening sound effects with progression of their condition “now it’s started to do it (crunch) down here as well” (as if my arm is above my head) (I/V15).

### Impact

The impact that a known rotator cuff tear has upon activities of daily living, leisure activities and occupation were described by patients. The emotional impact, financial impact and impact upon caring roles described by participants and their lives were also emphasized and are presented.

#### Activities of daily living

In consequence to the symptoms described, participants experienced significant and adverse impact upon their daily activities associated with their rotator cuff tears, “it did become very restrictive as to what I could do and couldn’t do (I/V1). Activities, if continued, took longer, for example a task which “would’ve taken me like an hour, it took me nearly a whole day (I/V2). The majority of participants described how simple daily activities, such as washing and dressing, lifting, carrying, reaching, filling up the kettle, driving, reaching for wallet, became either impossible or difficult. This was particularly the case if the tear affected the dominant arm (I/V19, 20) and eased if the participant had a non-affected “good arm” (I/V9) to use instead. One participant offered this description explaining how activities, even those jobs previously disliked, became impossible:

“I’ve always done things, I’ve done what I want to do and then suddenly that element’s taken out. What do I do?.....I can’t even do *boring* things that I hate that my wife likes me to do!.........I was bored witless…….And that (my usual life) was *gone.* It drove me up the wall.” (I/V 6).

#### Leisure

Rotator cuff tears also negatively influenced participant’s leisure activities. Participant’s halted leisure activities if advised to by health care professionals “I used to do an awful lot of cross stitch, she told me not to do that” (I/V1), or if activities became impossible “I couldn’t play tennis, absolutely out of the question, I couldn’t even lift the racquet up” (I/V11). People who had played sport “for decades” (I/V11) found the inability to continue these was hugely detrimental. One participant, who had played golf with his friends for over 30 years, spoke of losing this, and the time with his friends, and how he was reduced to spending his time doing jigsaw puzzles alone instead (I/V 6). Returning to golf, after he later had surgery, was immensely valued. Some people found that they could retain their leisure activities with the assistance of others, e.g. to land their fish during fishing (I/V 2). Fishing was the most frequently affected activity described by male participants in this study I/V 2,9,12) because of the need to cast the line, but as the cross stitch example indicates, both sporting and non-sporting leisure activities could be affected.

#### Occupation

The majority of participants were retired, unlike the earlier study by Nyman et al [12] whose younger participants emphasized impact upon work more specifically. Of those who worked, several were working part time due to their shoulder plus other co-morbidities, which placed them under financial pressures “I need to work more hours and……I physically can’t” (I/V 3). This participant worked in a supermarket and believed that shoulder problems were worse than back/mobility problems because people with sticks/wheelchairs “can operate the tills” whereas shoulders impact upon every job and employers “get sick of you”. This unseen nature of shoulder pain also affects patients with frozen shoulders [15]. Another participant felt unwillingly stuck in the house until he “stumbled across” voluntary work in a charity shop where, unlike his experience with various employment agencies, he was met with an attitude he considered helpful and problem solving:

“I can’t do a lot because of my shoulder……..”well” she said “I’m sure we can get over that” So they bought me a wheely trolley” (I/V 2)

The success of this lead to subsequent part time paid employment in the same organisation. For the few participants who were self-employed, rotator cuff tears could cause business worries, “It was quite stressful because of the worry…..what is going to happen to the business (I/V 12) and “I had a lot of work on at the time and I was trying to get through that” (I/V 20). One participant and his business partner took out additional sickness insurance following, and solely due to, his experience of rotator cuff tear.

#### Emotional impact

The majority of participants provided vivid descriptions regarding how rotator cuff tears, particularly experienced pain, had profound adverse emotional impact upon their lives:

“it (shoulder) just wears you down…..I just got so down about it. It was awful. So she (GP) put me on amitriptyline…….it was not a good time in my life” (I/V1)

“it was killing me….it was destroying me” (I/V2)

Participants were “desperate” (I/V3) to get their shoulder fixed:

“I was getting to the point where I thought I might take a lot of tablets and just not bother waking up, yeah I did get that bad” (I/V3).

This strength of expressed feeling by some participants is beyond that found in previous shoulder studies [12,15]. It is starting to be recognised that depression and anxiety negatively impact upon outcome after rotator cuff repair [22] and require greater attention from clinicians. In addition to being “depressing” (I/V 3, 15) and feeling “awful” (I/V6) many participants felt they became “edgy, short tempered” (I/V5) and “horrible to live with” (I/V 14) due to pain and lack of sleep. One participant spoke about re-injuring his shoulder when he thought it was improving and the realisation that “I really can’t deal with this anymore” (I/V13). Another spoke of feeling “dragged down when you have a pain that won’t go away (I/V 19) whilst another found the impact of reduced function “very frustrating and extremely unhappy” (I/V18). Several participants felt “people don’t believe you” (I/V 3), generally this did not refer to close family members but health care professionals or work colleagues or people known socially (I/V3, 14).

#### Finance

In addition to the financial impact of being unable to work at all/full time, a few participants had spent money on private health care. This was predominantly viewed negatively because, for these participants, consultations and treatments hadn’t worked hence their referrals to hospital orthopaedic appointments:

“so for a year, and an awful lot of my money, I got treatment for a trapped nerve” (I/V2)

“to try and get it (shoulder treatment) quick, I went private…..he (surgeon) gave me a minimal examination and didn’t really talk to me…….he charged me quite a lot of money for being in his office for 10 minutes” (I/V20).

The exception to this was one participant whose previous good experience with his physiotherapist, meant he didn’t hold it against them when diagnosis and treatment was unsuccessful on this occasion (I/V 11).

#### Social support

Participants mentioned how living with a rotator cuff tear was assisted by having a partner/carer who could help them do activities such as using “can openers and peeling potatoes” (I/V 3). However, if the participant was the carer for someone else, then the ramifications and impact of a rotator cuff tear could impact upon their ability to continue their caring roles. Two participants had spouses who used wheelchairs, and manoeuvring the chair up kerbs and on uneven ground became problematic. As one participant put it, a symptomatic rotator cuff tear “it’s got to affect the whole family, not just the person that it is happening to” (I/V15).

### Coping strategies

#### Getting on with life

Many participants attempted to cope by carrying on as normally as possible for as long as possible despite pain and problems “I got on the best way I could “(I/V 1), “I still carried on….it was being used as best as I can” (I/V19) and “this (coping with tear) is a mind over matter business (I/V17):

“…all of that (my activities) still proved really difficult and driving was horrendous. I still did it” (I/V 3)

The long term lived experience and nature of tears lead participants to believe that “if you still want to do things, you can’t let it (cuff tear) get in the way too much” (I/V 9).

#### Acceptance

Some participants spoke of their acceptance of their tear:

I’m not kidding myself here, (that) it’s all gone back and healed because I know that can’t happen. But I can manage it” (I/V17)

Another participant accepted that, as the body ages, the body’s ability to heal and function changes “it gets a bit depressing but I’m 68 and had a reasonably active life so I can’t complain” (I/V15). One participant however, spoke of how their shoulder had forced change upon them earlier than they wished:

“you’re getting older, you want to …..do physical things as long as you can. You know there’s a day when you’re not going to be able to do them but I wanted to keep going as long as I could. I couldn’t.” (I/V 6)

One participant though used distraction, rather than acceptance, to cope by using mediation and spending time with other people out and about (I/V 8). Overall however, there was a general view that people with rotator cuff tears had to appreciate the limitations of their shoulder now “it’s a matter of knowing how far you can go……..I know my limits” (I/V 17) and balancing getting on with life whilst restricting pain-provoking movements.

#### Dominance and the other shoulder

Participants whose dominant arm was affected understandably found this particularly problematic. Participants relied on using their other arm, if it was considered a “good” arm, to compensate when possible “at one time being left handed, everything was (done) right handed” (I/V 6), “I’ve got a good (other) arm” (I/V9). One participant, whose shoulder pain eased after many months, still avoided certain movements due to fear of re-injury “I will have to do it with the other arm, because I don’t want to hurt it (my shoulder)” (I/V17). Another participant recounted that, having learned to use a computer mouse in their “good” hand, they have continued this even though, again after many months, their rotator cuff tear pain has finally eased. The fear of re-injury or re-provoking the awful months of initial pain following rotator cuff tear has a continued impact on how participants move and use their shoulder subsequently. Another participant talked about giving their shoulder “more respect” now (I/V4). Several participants had previously had a tear in their other shoulder which influenced them in that they were actively keen to get their current tear sorted out as soon as possible (quicker than last time) because;

“I really don’t want to go through that again…it really was such a bad time, the pain was unbelievably bad” (I/V 1).

### Analgesia, aids and adaptions

Participants recognised the role that analgesia played in the management of their symptoms “to try and get rid of it (pain), I’d take my paracetamol” (I/V 6). But the need for long term pain relief, was considered a problem in its own right by a few participants. One participant was “trying not to take pain killers now because I’m losing my memory” (I/V 3), attributing this to long term analgesic use for her shoulder and other musculoskeletal conditions. Another few spoke of side effects related to analgesia such as headaches (I/V 19) and stomach problems (I/V 9). A heat pack was also used by one participant to try and ease the pain (I/V 6). Several participants mentioned they used a sling prescribed to some trial participants to help them “remember not to use it (shoulder) too much because then it’s painful” (I/V 1) and to rest their arm. One participant found this particularly helpful in early days of living with their tear, to rest their arm and avoid heavy work, and then weaned himself off the sling:

“over a matter of a couple of months I became a little more confident……..but it was a long long time before I’d risk picking the kettle up” (I/V 12)

Most participants knowingly and deliberately planned and modified how they carried out activities to enable them to continue with their activities, work and leisure pursuits. Examples included swapping bags to the other shoulder and taking a trolley shopping (I/V 8), going coarse fishing rather than fly fishing (I/V9), altering dance moves so partner’s went round each other rather than under raised arms (I/V9), pacing work activities (I/V 14), thinking ahead and planning movements so they don’t hurt/aggravate (I/V 17), using a computer mouse in their other hand (I/V 9), using hand-outs in classes rather than writing on a whiteboard (I/V 19). This approach was summed up by one participant as:

“I am still physically active, I still do what I can, but I’m very careful what I do” (I/V 17).

## Discussion

The intensity of symptoms and wide-ranging impact of symptomatic rotator cuff tears on all areas of life were described by study participants. Like frozen shoulder, painful shoulders with rotator cuff tears are hugely disruptive to people’s lives [15]. This supports the validity of the use of some or all of these components; pain, emotional, work, social, in well-designed Patient Related Outcome Measures (eg. DASH [23,24], WORC [25], RCQoL [26],OSS [27,28]) used in shoulder studies. These measures seek to quantify what is ‘heard’ qualitatively in this study. The Study participants described rotator cuff pain, and its impact, differently than people with other long term musculoskeletal pain conditions. Chronic pain is often described as episodic and unpredictable in nature, with ‘good days and bad days’ [29]. Here, study participants spoke about learning consequences: certain specific movements/activities were perceived as causing certain symptoms (predominantly pain) for a certain time period. Participants weighed up cause and effect in a balancing act that was often conscious “If I do X movement/activity then I will suffer Y in consequence for Z time”. This provided participants with an element of choice; comments such as “I know my limits” and let participants decide whether to remain within their limits or knowingly choose to do something risky or pain provoking. The question arises whether shoulder patients are able to link activity to symptoms in limbs, in a way that patients with central/torso pain cannot achieve, and thus avoid provoking pain by modifying movements. If symptoms changed over time, patients usually described adapting the balancing process accordingly. The exceptions to this seemed to be either movements/activities which participants had adapted so successfully that they continued them (such as using a computer mouse in their other hand), or movements/activities that participants perceived as remaining so risky and unsafe that they avoided them to lessen the risk of further/future cuff tears (eg reaching behind to pick up something on the back car seat).

### Limitations

Participants in this study had all been referred to hospital orthopaedic departments due to the severity of their condition. It has previously been demonstrated that patients with higher functional disability have lower quality of life [30]. The experiences of people with less severe symptoms, or whose symptoms have settled/eased over time or whose tears have responded well to treatment in primary care may be very different. Additionally, interviews were taken part after participants had completed their participation in the UKUFF trial; the time delay and any subsequent treatments may retrospectively have influenced or nuanced participants’ views.

### Clinical implications

Rotator cuff pain is associated with cuff degeneration and aging [1] yet the demands for higher levels of shoulder function later in life are increasing, due to factors such as the rising UK retirement age and the continuation of occupational and leisure activities later in life. Recently, the mismatch between clinician’s and patients perceptions of shoulder pain was highlighted [15]. As previously with frozen shoulder, people living with rotator cuff tears described these in terms of a ‘biographically disruptive event [15,31] which clinicians need to recognise and fully appreciate.

## Conclusions

Clinicians need to appreciate and understand the intensity and shocking nature of pain that may be experienced by participants with known rotator cuff tears and understand the detrimental impact tears can have upon all areas of patient’s lives. Clinicians also need to be aware of the potential emotional impact caused by cuff tears and to ensure that patients needing help for conditions such as depression are speedily identified and provided with support, explanation and appropriate treatment. Conservative and surgical treatments of symptomatic rotator cuff tear aim to relieve pain, however, further research concerning the management of pain for this patient group appears indicated by this study.

## Competing interests

The authors declare that they have no competing interests.

## Authors’ contributions

All authors contributed to the design and co-ordination of the study. Catherine Minns Lowe carried out the interviews, led the data analyses and drafted and revised the manuscript. Jane Moser peer reviewed the data analyses and critically commented upon the important intellectual content of the manuscript. Karen Barker independently coded transcripts, peer reviewed the data analyses, was responsible for the study’s Research Governance and critically commented upon the manuscript. All authors read and approved the final manuscript.

## Pre-publication history

The pre-publication history for this paper can be accessed here:

http://www.biomedcentral.com/1471-2474/15/228/prepub
